# Molecular characterization of pediatric mastocytosis revealed different somatic mutations with uncertain prognostic value

**DOI:** 10.3389/fcell.2026.1780799

**Published:** 2026-02-26

**Authors:** Deborah Kasmi, Fiorina Giona, Michela Ribersani, Giorgio Sforzini, Massimo Breccia, Giovanna Palumbo

**Affiliations:** Hematology, Department of Translational and Precision Medicine, Policlinico Umberto I- Sapienza University, Rome, Italy

**Keywords:** children, cutaneous mastocytosis, mutational landscape, next-generation sequencing (NGS), polymerase chain reaction (PCR), prognosis

## Abstract

**Background:**

Mastocytosis is a rare clonal hematological neoplasm, characterized by cutaneous manifestations in children and categorized as: maculopapular cutaneous mastocytosis (MPCM), diffuse cutaneous mastocytosis (DCM) and mastocytoma. Systemic mastocytosis (SM) typically occurs in adults with c-*KIT* D816V mutation. Additional genetic mutations (*TET2*, *NRAS*, *SF3B1*, *ASXL1*, etc.) have been detected using Next-Generation Sequencing (NGS) in the adult population while limited information is available in the pediatric setting.

**Methods:**

36 patients (pts) with pediatric mastocytosis diagnosed between 1997 and 2021 were included. Peripheral blood samples were collected to detect c-*KIT* D816V mutation, using both RT-PCR and ddPCR techniques, and to investigate other molecular mutations using NGS panel for rare and myeloid genes.

**Results:**

Median age of lesion onset was 4.7 months (range birth-17.8 years). 58% of the cohort underwent cutaneous biopsy after a median 3.77 months from lesion onset (range 2.49 months–11.6 years). 20 (55%) were classified as MPCM, 10 (28%) as DCM and 6 (17%) as mastocytoma. Median tryptase value at the onset was 5 ng/mL: MPCM (range 1.2–141 ng/mL) vs. DCM (range 2.71–19.4 ng/mL) vs. mastocytoma (range 3.8–7.3 ng/mL). Two MPCM pts developed indolent SM (ISM) after 10 and 20 years from the onset of disease. RT-PCR identified c-*KIT* D816V mutation in 4 pts (2 MPCM, 1 DCM, 1 ISM). NGS revealed the precedent mutation in 3 pts, c-*KIT* D816Y and c-*KIT* Y553C in 2 pts. An additional 10 myeloid gene mutations were detected by NGS: 5 already known (*ASXL1* G1397S; *JAK2* L393V; c-*KIT* D816Y; *LNK* E208Q; *TET2* Y867H) and 5 not previously described (*ETV6* A215P; c-*KIT* Y553C; *NFE2* I291T; *SH2B3* G382D; *SH2B3* L438V). A single mutation was found in 7 pts (3 MPCM, 3 DCM, 1 ISM), while two or more mutations in 3 DCM pts. Overall, 9/36 pts (5 DCM, 3 MPCM, 1 mastocytoma) presented spontaneous complete regression of cutaneous lesions after a median time of 25 months (range 17 months–25 years).

**Conclusion:**

c-*KIT* mutations resulted in 35% of the children tested. The RT-PCR technique resulted more sensitive in finding c-*KIT* D816V, while NGS in detecting other mutations whose prognostic roles require further investigation.

## Introduction

Mastocytosis is a rare heterogeneous disease characterized by a pathological proliferation and activation of morphologically and immunophenotypically abnormal mast cells (MCs) in various tissues, particularly in the skin (cutaneous mastocytosis–CM) and systemic organs (systemic mastocytosis–SM) such as the bone marrow, liver, spleen and the lymph nodes (Valent, 2013; Akin and Valent, 2014; Valent et al., 2017). The estimated prevalence of CM is 1–3 per 10,000, while the prevalence of SM is approximately 1 per 10,000 (Valent et al., 2023). Generally, most pediatric patients with CM, are associated with a tendency for spontaneous regression around puberty and a favorable prognosis (Hartmann et al., 2016; Lange et al., 2021; Tiano et al., 2023; Renke et al., 2023; Schaffer, 2021). According to the international consensus statement, characteristic presentations of CM are maculopapular cutaneous mastocytosis (MPCM), with two variants, namely, monomorphic (mMPCM) and polymorphic (pMPCM), diffuse cutaneous mastocytosis (DCM), and cutaneous mastocytoma (MS) with isolated (iMS) or multilocalized (mMS) form (Hartmann et al., 2016; Arber et al., 2022; Khoury et al., 2022). Despite the fact that the clinical phenotype of childhood-onset mastocytosis is different, and that the morphology of skin lesions may change during the course of the disease, a pathognomonic feature of all forms is a positive Darier’s sign, which presents as a wheal and-flare reaction, which occurs within a few minutes after stroking a CM lesion (Hartmann et al., 2016; Lange et al., 2021; Ługowska-Umer et al., 2023). Among the mediator-related manifestations, pruritus is the most reported symptom in this population (Hartmann et al., 2016; Lange et al., 2021; Ługowska-Umer et al., 2023; Polivka et al., 2021; Giona, 2021). Late-onset of the disease in pediatric age, monomorphic clinical form, persistence of skin lesions after puberty, organomegaly, and significant abnormalities in the complete blood count are the predictors of SM (Tockova et al., 2025). The latter is present mainly in indolent form (ISM), whereas advanced pediatric SM is very rare. Unlike adults, almost all children with systemic disease have skin lesions with mMPCM as the most common type of skin features (Lange et al., 2021; Schaffer, 2021; Brockow et al., 2025; Carter et al., 2018).

c-KIT is a tyrosine kinase receptor expressed on the surface of MCs, melanocytes, germ cells, hematopoietic stem cells and gastrointestinal stromal cells. The dimerization of c-KIT by stem cell factor impacts MC proliferation and differentiation. Somatic gain-of-function point mutations within the c-KIT gene play a crucial role in developing clonal disorders. In addition to being a minor criterion for diagnosis, identifying and quantitating the c-*KIT* p. D816V mutation in peripheral blood leukocytes (PBLs) became a reliable predictor of SM in adults (Brockow et al., 2025; Kristensen et al., 2020). A similar approach was recently taken regarding pediatric patients; the c-*KIT* p. D816V mutation in PBLs of children was positive in most patients with SM, while it was not identified in the PBLs of children known to have only cutaneous disease (Tockova et al., 2025). This study suggested that blood mutation determination should be considered in the diagnostic work-up for pediatric mastocytosis, particularly in rare cases with suspected SM. Additional genetic mutations associated with myeloid malignancies involving (*TET2*, *NRAS*, *SF3B1*, *ASXL1*, etc.) have been detected using Next-Generation Sequencing (NGS) in adults with mastocytosis. Limited information is available regarding the use of this method in patients diagnosed in pediatric age. This study focuses on identifying patients (pts) with different cutaneous forms at risk of developing aggressive systemic disease, detecting the known c-*KIT* D816V mutation using RT-PCR, identifying different c-*KIT* and/or additional gene mutations using NGS, and evaluating the potential role of different mutations in the outcome of pts with mastocytosis, aged <18 years at the onset of disease.

## Materials and methods

### Materials

Mas_Ped1 was a single-center observational, retrospective-prospective study supported by the Italian Mastocytosis Association (ASIMAS). It enrolled patients diagnosed with cutaneous or systemic mastocytosis, aged <18 years at diagnosis, at the Hematology Department of “Sapienza” University of Rome from April 1997 to February 2021. The present investigation was conducted in compliance with ethical principles and good clinical practice (GCP) to protect the rights, safety, and wellbeing of participating patients, in accordance with Ministerial Decree No. 162 of 15 July 1997, and subsequent updates. The study was approved by the Ethics Committee of the Policlinico Umberto I/Sapienza University of Rome in March 2020. Only after receiving detailed explanations about the nature and purposes of the study, patients and/or their parents/guardians would sign the consent forms for participation in the study, appropriate for the subject’s age: one form for children between 6 and 12 years old and one for adolescents between 12 and 17 years old. For children <6 years old, a consent form was required for parents only. A specific form was required for patients diagnosed with pediatric mastocytosis but ≥18 years old at the time of the study.

### Methods

The following clinical and laboratoristic data were taken into consideration: family history of hematological disorders; comorbidities; age at onset of disease and at diagnosis; number, location, and type of skin lesions according to the 2016 WHO criteria; hemoglobin, leukocytes, platelets, ferritin, and transferrin; possible presence of cytopenias; serum tryptase (considered normal values: <11 ng/mL); evaluation of any abdominal organomegaly (liver and spleen), lymphadenopathy, and bone lesions; information on antihistamine therapy and any previous anaphylactic episodes. Genetic analyses and NGS on peripheral venous blood were performed on samples collected on-site and then sent by courier to the laboratories of the institutions with which we collaborate.

*FIP1L1*-*PDGFRA s*earching was conducted at hematology-focused Internal Medicine laboratories at the University Hospital San Luigi Gonzaga Orbassano Turin. The technique used was a nested RT-PCR. The molecular investigations using NGS were performed at the CRIMM (Research and Innovation Center for Myeloproliferative Diseases) of the University Hospital Careggi in Florence with a technique reported elsewhere (Buermans and den Dunnen, 2014). The analysis was performed using Ion Reporter software; the reference genome used was the Human Reference Genome version hg19. The myeloid lineage rearrangement panel includes 27 genes: *ASXL1* (exon 12), *CALR* (exon 9), *CBL* (exons 8 and 9), *KIT* (exons 8–11, 13, 17, 18, 19), *CSFR3* (exons 7, 8, 13–17), *CUX1* (exons 4–6, 9, 15–19, 21–23), *DNMT3A*, *ETNK1* (exon 3), *EZH2*, *IDH1* (exon 4), *IDH2* (exon 4), *IKZF1* (exons 3–5, 8), *JAK2*, *KRAS*, *MPL*, *NFE2* (exon 3), *NRAS*, *PTPN11* (exons 3, 13), *RUNX1*, *SETBP1* (exon 4), *SF3B1* (exons 12–16), *SH2B3* (exons 2, 3, 4), *SRSF2* (exon 1), *TET2*, *TP53*, *U2AF1*, *ZRSR2*. Regarding the identification of the c-*KIT* D816V variant, NGS is not sufficiently sensitive. Thus, the findings were confirmed by real-time PCR analysis, especially when detected below the analytical threshold of 5%. For the specific and accurate evaluation of this variant, the reference method outlined in the document D/2281L/3-Table Laboratory Tests CRIMM was employed (AOU Careggi, 2023). Variant Allele Frequency (VAF) detection limits in real-time PCR (qPCR) assays are highly sensitive, typically capable of identifying variants with a VAF as low as 0.003%–0.01%. High-sensitivity allele-specific qPCR (ASO-qPCR) and droplet digital PCR (ddPCR) methods when utilized can detect very low amounts of mutated cells with a VAF ≤0.01%. The panel for rare rearrangements includes 29 genes: *ABL1*, *ALK*, *BLC2*, *BRAF*, *CCND1*, *CREBBP*, *EGFR*, *ETV6*, *FGFR1*, *FGFR2*, *FUS*, *HMGA2*, *JAK2*, *KMT2A* (*MLL*), *MECOM*, *MET*, *MLLT10*, *MLLT3*, *MYBL1*, *MYH11*, *NTRK3*, *NUP214*, *PDGFRA*, *PDGFRB*, *RARA*, *RBM15*, *RUNX1*, *TCF3*, *TFE3*. For each sample, the minimum acceptance requirements for the analysis correspond to an average coverage of ≥1,000X, a minimum target coverage of 95%, and a minimum read count of 250. Variants with a VAF of ≥5% are identified. All regions with coverage less than 50X are excluded from the analysis. Variant analysis for functional prediction and population frequency was performed by consulting the public databases COSMIC, dbSNP, GnomAD; *in silico* functional prediction tools (SIFT, PolyPhen2, Mutation Taster, FATHMM, CADD), and GERP++. For splice variants, the Human Splicing Finder tool was consulted.

## Results

Thirty-six patients were included in our study. Of the 36 patients, 19 were male (53%) and 17 were female (47%). All patients had a form of exclusively cutaneous mastocytosis at the time of admission. Specifically, 20 patients (55%) had MPCM, 10 patients (28%) had DCM, and 6 patients (17%) had mastocytoma (MS). Of the 20 cases of MPCM, 13 (65%) were male and 7 (35%) were female; of the 10 cases with DCM, 2 were male (20%) and 8 were female (80%); of the 6 patients with mastocytoma, 4 were male (67%) and 2 were female (33%). The M/F ratio is 1.12 in the overall population, 1.85 in MPCM, 0.25 in DCM and 2 in MS. The median age at onset of skin lesions was 4.7 months in the overall population (range birth-17.8 years), 4.8 months for MPCM cases (range 0.56–7.7 years), 5.7 months for DCM (range birth-17.8 years), and 3.6 months for mastocytoma (range 1.6–22.5 months). Twenty-five patients (69%) had their first lesions within 1 year of age, eight patients (23%) between the ages of 2 and 10, and three patients (8%) between the ages of 10 and 18. Age at onset and diagnosis of patients categorized by type of skin lesions are displayed in Table 1. The median age at diagnosis is 8.5 months in the population (range 3.0 months–27.9 years), 10.8 months (range 3.0 months–27.9 years) for MPCM, 31.3 months (range 5.1 months–18 years) for DCM, and 5.2 months (range 3.9–22.5 months) for MS. Nineteen patients (53%) had their first lesions within 1 year of age, 14 patients (39%) between the ages of 2 and 10 years, and 3 patients (8%) between the ages of 10 and 18 years.

**TABLE 1:** Age at onset and diagnosis of patients categorized by type of skin lesion.

Variables	Total	MPCM	DCM	Mastocytoma
Patients, n	36	20	10	6
Median age at onset of lesion				
(months) (median/range)	4.7 (0–17.8 years)	4.8 (0.56–7.7 years)	5.7 (0–17.8 years)	3.6 (1.6–22.5 years)
Class of age at onset of lesion (%)				
<1 year	25 (69%)	14 (70%)	6 (60%)	5 (83%)
2–10 years	8 (23%)	4 (20%)	3 (30%)	1 (17%)
>10 years	3 (8%)	2 (10%)	1 (10%)	0
Age at diagnosis				
(months) (median/range)	8.5 (3.0–27.9 years)	10.8 (3.0–27.9 years)	31.3 (5.1–18 years)	5.2 (3.9–22.5)
Class of age at diagnosis (%)				
<1 year	19 (53%)	10 (50%)	4 (40%)	5 (83%)
2–10 years	14 (39%)	8 (40%)	5 (50%)	1 (17%)
>10 years	3 (8%)	2 (10%)	1 (10%)	0
Time between onset lesion and diagnosis				
(months) (median/range)	3.77 (2.49–4.09 years)	6.08 (3.05–41.2)	25.61 (2.49–4.09 years)	1.63 (3.21–46.5)

DCM, diffuse cutaneous mastocytosis; MPCM, maculopapular cutaneous mastocytosis.

Clinical and laboratory features of the patients analyzed are shown in Table 2. Of the 36 patients, 10 (28%), including 5/20 with MPCM (25%), 4/10 with DCM (40%), and 1/6 with mast cell tumor (17%), had a family history of hematological disorders. In detail, 3 patients had a family history of leukemia, 1 of hemochromatosis, 1 of hemophilia A, 1 of thalassemia major, 1 of Monoclonal Gammopathy of Undetermined Significance (MGUS), and 1 of lymphoma. Two sisters with mastocytosis had a family history of favism.

**TABLE 2:** Baseline patient characteristics.

Variables	Total	MPCM	DCM	Mastocytoma
Patients, n	36	20	10	6
Family history of hematological diseases, n (%)	10 (28%)	5 (25%)	4 (40%)	1 (17%)
Comorbidities, n (%)	13 (36%)	9 (45%)	2 (10%)	2 (33%)
Hepatomegaly, n (%)	0	0	0	0
Splenomegaly, n (%)	1 (3%)	0	1 (10%)	0
Hemoglobin, (g/dL) median (range)	12.7 (9.2–17.1)	12 (9.2–15.3)	13 (11.9–14.7)	13.4 (11.4–17.1)
WBC count, (× 10^9^/L) median range	7.8 (4.5–15.6)	7.3 (4.5–15.6)	7.5 (5.4–9.4)	9 (6.5–14.4)
PLT count, (× 10^9^/L) median range	311 (191-678)	322 (191–678)	304 (222-458)	281 (192-474)
Tryptase, (ng/mL) median range	5 (1.2–141)	4.97 (1.2–141)	5 (2.71–19.4)	5.45 (3.8–7.3)
Skin biopsy patients, n (%)	21 (58%)	13 (65%)	6 (60%)	2 (33%)

DCM, diffuse cutaneous mastocytosis; MPCM, maculopapular cutaneous mastocytosis; PLT, platelet; WBC, white blood cell.

**FIGURE 1:**
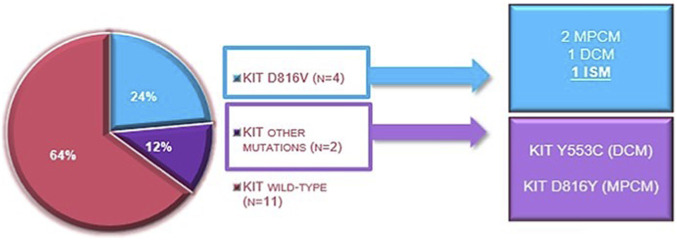
Identified c-*KIT* mutations in the whole cohort alongside the number of cases and the associated variants of mastocytosis; DCM, diffuse cutaneous mastocytosis; ISM, indolent systemic mastocytosis; MPCM, maculopapular cutaneous mastocytosis.

Of the 36 patients, 13 (36%), including 9/20 with MPCM (45%), 2/10 with DCM (20%), and 2/6 with MS (33%), had comorbidities. A patient had developed MPCM at 8 years of age, in remission and off therapy from a previous acute B-lymphoid leukemia with a transcript TEL/AML1. A patient with thalassemia major and pharyngeal xanthogranuloma had developed MPCM since the age of 5. Immunologically based disorders were found in four patients: celiac disease (2 patients, 1 with DCM and 1 with MS), atopic dermatitis (1 patient with MPCM), and multiple food allergies (1 patient with MPCM). One of the 2 patients, who also had celiac disease and mastocytoma, developed myofibromatosis at the age of 4, with two osteolytic lesions on the arm and one on the parietal area. None of the patients, including those with immune-related comorbidities, experienced episodes of anaphylaxis. The mothers of two patients with MPCM had gestational diabetes during pregnancy, resulting in normal infants at birth. One patient with mastocytoma was macrosomic at birth. In one case of MPCM, the mother presented hypothyroidism during pregnancy. Regarding clinical examination, no organomegaly was observed in any patient, except for one patient with DCM who presented with transient splenomegaly attributed to a self-limited viral infection. Blood count was evaluable in all patients. The median hemoglobin value in all patients was 12.7 g/dL (range 9.2–17.1 g/dL); similar in all the different forms. Anemia (Hb at diagnosis 9.2 g/dL) was present in one patient with MPCM and thalassemia major. The median leukocyte counts in all patients studied was 7.83 × 10^9^/L (range 4.5–15.6 × 10^9^/L) again similar among all the different forms. The median platelet counts in the patients analyzed was 311 × 10^9^/L (range 191–670 × 10^9^/L) without any differences considering the different forms analyzed. Tryptase was detectable at diagnosis in 33 patients. The median tryptase value was 5 ng/mL, with a range of 1.2–141 ng/mL. Considering the different forms, the median tryptase level in MPCM was 4.97 ng/mL (range 1.2–141 ng/mL), 5 ng/mL (range 2.71–19.4 ng/mL) in DCM, and 5.45 (range 3.8–7.3 ng/mL) in mastocytoma. Of 36 patients, 21 (58%) underwent a skin biopsy to confirm the diagnosis of mastocytosis. During follow-up, due to clinical suspicion of a systemic form, 4 patients (11%) underwent a bone marrow biopsy, which allowed the diagnosis of 2 indolent forms of SM from an exclusively cutaneous MPCM. The first one had a medullary CD117+ mast cell component of 40%, while in the second one it was 15%. In the other 2 patients (1 MPCM and 1 DCM), the CD117+ mast cells were <10%. Based on the 2016 WHO criteria, 2 patients (6%), both male, with MPCM, diagnosed at 15 and 18 years of age, respectively, were considered to have progressed to indolent systemic mastocytosis (ISM), at 25 and 38 years of age, respectively. The progression occurred 10 years after the onset of skin lesions in the first patient, and 20 years in the second. PCR testing for mutations in the c-*KIT* oncogene was performed in 17/36 patients (47%) and was positive in 4/17 patients (24%), with the “classic” c-*KIT* D816V mutation. Considering the forms that emerged from the re-evaluation during follow-up, the c-*KIT* D816V mutation was found in 2 patients with MPCM, 1 patient with DCM, and 1 patient with ISM. All 17 patients investigated with the PCR method were re-evaluated using NGS. With this method, the c-*KIT* D816V mutation was found in 3 of the 4 patients who had been found to have the mutation by PCR, and other c-*KIT* mutations were identified, namely, the c-*KIT* Y553C mutation in one patient with DCM and the c-*KIT* D816Y mutation in one patient with MPCM (Table 3). Considering the type of mastocytosis, of the 6 patients with c-*KIT* gene mutations, 3 had a cutaneous MPCM form, 2 had a cutaneous DCM form, and 1 had an ISM (Figure 1).

**TABLE 3:** Identified c-*KIT* mutations using PCR and NGS techniques.

c-*KIT* status	PCR	NGS
*c-KIT* D816V	4	3
*c-KIT* other mutations	0	2
*c-KIT* wild-type	13	12
TOTAL	17	17

NGS, next-generation sequencing; PCR, polymerase chain reaction.

**FIGURE 2:**
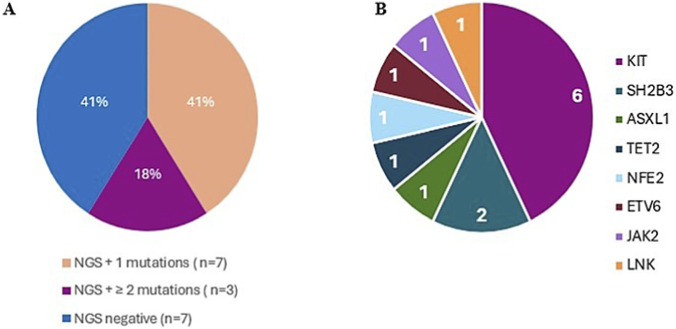
Pie charts illustrating NGS identified mutations in the whole cohort. **(A)** Classification of patients according to number of mutations detected. **(B)** Altered genes alongside the number of cases detected.

**FIGURE 3:**
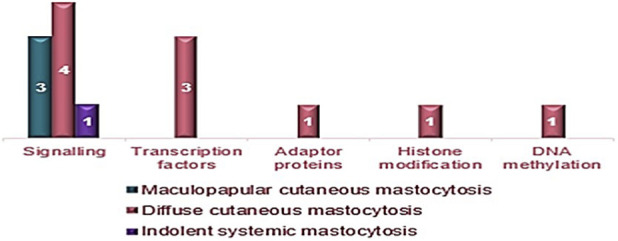
Functional groups of identified mutations according to the variant of mastocytosis.

Of the 17 patients examined by NGS, 10 (59%) had at least one mutation in either a myeloid or a rare gene (Figure 2). Most patients (7/10) had a single mutation, while 3 patients with the DCM form had two or more mutations. 11 different mutations were detected. Of these mutations, 5 were identified for the first time in this setting, while 6 were known and associated with other hematological diseases and with known clinical significance. Of these 6 mutations, only 2 had previously been associated with mastocytosis. The corresponding list is available in the Supplementary Table S1. Considering the functional group of mutations found by NGS, including c-*KIT* mutations, the largest number of mutations (8/14, 58%) involved intracellular signaling. The most frequent mutations involved transcription factors (*ETV6*, *NFE2*, *JAK2*, each in one patient) and the signaling functional group (*SH2B3* in two patients). The less frequent mutations were those affecting genes in the histone modification group (*ASXL1*), adaptor proteins (*LNK*), and DNA methylation (*TET2*), each found in one patient. Regarding the functional groups in the different cutaneous forms, it emerged that 10/14 mutations (72%) are attributable to DCM, 3/14 (21%) to MPCM, and 1/14 (7%) to ISM (Figure 3). Considering the functional group of the mutations found and the PCR results, it emerges that the only mutations associated with the c-*KIT* D816V mutation are one for the histone modification group (*ASXL1*) and one for the transcription factors group (*JAK2*). Six mutations found by PCR are not associated with the c-*KIT* D816V mutation: they concern genes involved in signaling (*SH2B3* in 2 patients), in the synthesis of transcription factors (*NFE2*, 1 patient; *ETV6*, 1 patient) or adaptor proteins (*LNK*, 1 patient) or in DNA methylation (*TET2*, 1 patient) (Table 4). Considering the progression of lesions in the 10 patients with mutated genes by NGS, three patients with DCM, with mutations in *NFE2* I291T (1 patient), *SH2B3* G382D + *ETV6* A215P (1 patient), and *ASXL1* G1397S + *JAK2* L393V + c-*KIT* D816V (1 patient), showed complete regression of the skin manifestations. As for the tryptase values in the 10 patients with mutations, the patients with the highest tryptase values were: one patient with c-*KIT* D816V mutation who evolved into an indolent systemic form (tryptase = 26 ng/mL), one patient with the c-*KIT* D816Y mutation and MPCM, present since birth (tryptase = 158 ng/mL) (Table 5). Of the 10 patients with mutations, 3 (2 females and 1 male) with DCM had multiple mutations. None of these patients had a systemic evolution of the disease; in fact, 2 showed spontaneous regression of the skin lesions at the ages of 10 and 22, approximately 9 and 4 years after their onset, respectively. Regarding treatment, all patients followed and continue to follow a histamine-free diet, eliminating foods such as chocolate, shellfish, and nuts. Anti-mast cell mediator therapy was prescribed as needed or as long-term symptomatic treatment in 58% of patients. In two-thirds of cases, a single drug was sufficient to relieve symptoms, and only a minority of patients required combination therapy. Ten patients were on chronic monotherapy, while 5 patients on polytherapy. The most prescribed drug was cetirizine: 6 patients used it as a single agent, and 3 patients were given it in combination with other drugs. Of the 36 patients, 3 patients (8%), 2 of whom were DCM and 1 MPCM, were lost to follow-up. The median follow-up of the 33 evaluable patients is 25.6 months, with a range from 4.3 months to 23.2 years. Overall, 9 patients (25%), 5/10 with DCM, 3/20 with MPCM, and 1/6 with mastocytoma, had complete regression of the skin lesions. In 5 of them (56%), the disappearance occurred between 2 and 10 years, in 2 between 10 and 18 years, and in another 2 over the age of 18 years. A particular trend of the lesions was observed in a patient with MPCM that began at 3 months of age, who had regression of the lesions at 18 years of age and a reappearance of the same at 24 years of age. Table 6 shows the temporal trend of tryptase levels, from the time of diagnosis to the last follow-up (median and range), considering all patients and dividing them into the different forms of CM. According to Alvarez-Twose proposed tryptase values defining 3 different patient categories: a) 6.6 ng/mL for initiating daily anti-mediator therapy; b) 15.5 ng/mL for hospitalization; c) 30.8 ng/mL for admission to intensive care, in our patients, the median tryptase value was <6.6 ng/mL in 20 patients (56% of the total), between 6.6 and 15.5 ng/mL in 9 cases (25%) and >15.5 ng/mL in 4 patients (11%) (Table 7). However, none of our patients apparently required hospitalization or admission to an intensive care unit.

**TABLE 4:** Spectrum of mutations per functional group in c-*KIT* D816V mutated patients.

Functional group	Gene	*c-KIT*	
		*c-KIT* D816V mutated N° patients	*c-KIT* D816V wild-type N° patients
Signalling	*SH2B3*	0	2
Histone modification	*ASXL1*	1	0
DNA methylation	*TET2*	0	1
Adaptor protein	*LNK*	0	1
Transcription factors	*NFE2*	0	1
	*ETV6*	0	1
	*JAK2*	1	0
Total N° of mutations		2	6

**TABLE 5:** Mutations identified in the whole cohort of patients with relative median serum tryptase levels categorized by mastocytosis type.

Type of mastocytosis	Identified mutations	Median serum tryptase levels (ng/mL)
DCM	*NFE2* I291T	4.11
DCM	*SH2B3* G382D + *ETV6* A215P	4.6
DCM	*ASXL1* G1397S + *JAK2* L393V + c-*KIT* D816V	3.73
ISM	*c-KIT* D816V	26
MPCM	*c-KIT* D816Y	158
DCM	*c-KIT* Y553C	9.36
DCM	*SH2B3* L438V	13.4
DCM	*TET2* Y867H + *LNK* E208Q	8.68
MPCM	*c-KIT* D816V	3.9
MPCM	*c-KIT* D816V	4.3

Patients with elevated tryptase levels are highlighted in light orange while those with regression of skin lesions in light blue.

DCM, diffuse cutaneous mastocytosis; ISM: indolent systemic mastocytosis, MPCM, maculopapular cutaneous mastocytosis.

**TABLE 6:** Temporal trend of tryptase serum levels according to cutaneous mastocytosis type.

Tryptase serum levels	Total	MPCM	DCM	Mastocytoma
Tryptase at diagnosis, (ng/mL) median range	5 (1.2–141)	4.97 (1.2–141)	5 (2.71–19.4)	5.45 (3.8–7.3)
Tryptase during follow-up, (ng/mL) media range	5.57 (1.6–181)	6.9 (1.6–191)	5.1 (2.8–12.6)	4.8 (4.4–5.44)
Tryptase of the last follow-up, (ng/mL) median range	5.7 (1.6–158)	6.9 (1.6–158)	5.5 (2.63–14.5)	4.8 (4.4–5.44)

DCM, diffuse cutaneous mastocytosis; MPCM, maculopapular cutaneous mastocytosis.

**TABLE 7:** Distribution ranges of median tryptase values in different forms of cutaneous mastocytosis.

Median tryptase value, n (%)	Total	MPCM	DCM	Mastocytoma
<6.6 ng/mL (%)	20/36 (56%)	10 (50%)	6 (30%)	4 (20%)
6.6–15 ng/mL (%)	9/36 (25%)	5 (56%)	4 (44%)	0
>15 ng/mL (%)	4/36 (11%)	4 (100%)	0	0

DCM, diffuse cutaneous mastocytosis; MPCM, maculopapular cutaneous mastocytosis.

## Discussion

In this analysis, no gender predominance was observed, while literature data highlight a slight female predominance in the subset considered (Matito et al., 2018). MPCM was found in 55% of patients, DCM in 28%, and MS in the remaining 17%. Published data report a significantly different distribution of CM forms in pediatric age. The percentage of MPCM is approximately 75%, DCM is less than 10%, while that of MS is approximately 15% (Hartmann et al., 2016). Another notable feature is the age differences at which lesions appear at diagnosis between the various skin forms. Specifically, the onset of all three variants typically occurs in the first months of life, with MS appearing earlier. A significant finding is that diagnosis occurred before the onset of lesions in MS (2 months) and MPCM (6 months), but not in DCM (2 years). Of the 36 patients with CM at onset, two developed ISM during follow-up: in one case, the diagnosis was made based on the presence of one major and one minor criterion, and in the other based on the presence of three minor criteria according to the 2016 WHO classification. In these patients, both asymptomatic, the presence of a neoplastic clone in the bone marrow (15% and 40% respectively), without peripheral cytopenia, and increased tryptase levels (values of 26 and 99.5 ng/mL, respectively) were detected. In both cases, the onset of the disease occurred later, just before the age of 18, with systemic evolution occurring at age 25 in one patient and at age 38 in the other. This would appear to confirm that patients with disease onset in adolescence are at greater risk of developing a systemic form. In our study, overall, of the patients whose lesions regressed, five had DCM, three had MPCM, and one had solitary mastocytoma. This finding is in line with the existing literature referring to DCM’s excellent remission rate 5 years after onset, although MPCM regresses with greater ease. Seven of these nine patients had a tryptase level <5 ng/mL at onset, with values at subsequent follow-ups remaining substantially in line. Of the remaining two patients, one never had a tryptase test performed, and the other had relatively low and consistent values over time (8.5 ng/mL at onset), indicating that low tryptase levels at onset and during follow-up are indicative of a high probability of disease remission. None of the cases whose lesions disappeared were anemic at presentation, while one had leukocytosis (white blood cells 13.26 × 10^9^/L) and thrombocytosis (platelets 678 × 10^9^/L) of a likely reactive nature. Patients with ISM had no abnormalities in their complete blood count or iron status.

PCR proved more sensitive in identifying the c-*KIT* D816V mutation than NGS, while NGS was able to identify other c-*KIT* mutations. This greater sensitivity of PCR in identifying the c-*KIT* D816V mutation is already noted in the literature for cases of ISM in adults (George et al., 2020). Moreover, Wetherby and colleagues have recently developed a highly sensitive digital PCR assay to this specific mutation with a VAF sensitivity of 0.005%, with the aim of using it as a simple tool to test peripheral blood in case of clinical suspicion of mastocytosis (Wetherby et al., 2023). The c-*KIT* D816V mutation has been found in 24% of all pediatric patients and has been identified in 29.5% of MPCM and 12.5% of DCM. Of note, one patient with MPCM with this alteration presented exalted serum tryptase levels (peaking at 191 ng/mL, almost twenty times the normal range. This mutation has already been described in adult systemic mastocytosis and is associated with the presence of a CD34+/CD117+ mast cell neoplastic clone (Beghini et al., 1998).

Five mutations (*SH2B3* G382D and *SH2B3* L438V, *ETV6* A215P, c-*KIT* Y553C, *NFE2* I291T) have been identified for the first time in this setting in four genes known to play a role in the pathogenesis of various hematological malignancies. Considering the functional group of mutations, 57% involved genes related to intracellular signaling (*KIT*-*SH2B3*), while 22% involved transcription factors (*JAK2*-*NFE2*-*ETV6*). Synthesis of intracellular adaptor proteins (*LNK*), histone chromatin modification (*ASXL1*), and DNA methylation (*TET2*) were each altered in 7% of patients. Two of the patients, harboring the following mutations *NFE2* I291T and *SH2B3* G382D associated with *ETV6* A215P, experienced skin lesions regression. Based on these elements, these alterations could potentially be associated with relatively early disease resolution. It also emerged that in the patient with the *SH2B3* L438V mutation, the median tryptase value was slightly above the normal (13.4 ng/mL, reaching a maximum value of 19.4 ng/mL). Somatic mutations of c-*KIT* are frequently found in mastocytosis and gastrointestinal stromal tumor (GIST), while its germline variants are rare, and only found in a few familial cases. c-*KIT* Y553C has been described as a germline mutation in GIST (Ke et al., 2016) and mucosal melanoma (Clavero-Rovira et al., 2024). Its significance in cutaneous mastocytosis has not been elucidated. The patient detected with this mutation showed neither disappearance of skin lesions nor elevated tryptase levels nor abnormalities in other biochemical parameters.

Most patients, however, had known mutations previously described. Many reported *ASXL1* (Additional sex combs like 1) gene mutations, which in SM are located at exon 12 and have been recurrently associated with a worse prognosis (González-López et al., 2022). G1397S alteration has been found in chronic myelomonocytic leukemia (CMML) and, although associated with increased cell proliferation, does not cause an effective reduction in overall survival (Patnaik et al., 2013). The patient with this mutation had DCM, tryptase levels of 3.73 ng/mL, and spontaneous regression of the lesions. Mutations in the JAK-STAT pathway have also been detected, but these alterations do not appear to have any clinical or prognostic significance. In the first case, *JAK2* L393V was localized in a non-coding region of the gene and, therefore not clinically relevant (Aranaz et al., 2010). The characteristics of the patient with this mutation have already been described for the *ASXL1* G1397S mutation, to which it was associated. Regarding *LNK* E208Q mutation detected in one patient with DCM, it appears to cause only partial loss of gene function and thus might not play a role in the pathogenesis of the disease (Oh et al., 2010). The patient with this mutation is currently clinically stable, without need for therapy, and has normal tryptase levels.

Additionally, *TET2* loss-of-function variants associated with c-*KIT* D816V mutations occur at a high frequency in SM (Tefferi et al., 2009). Rigo and colleagues demonstrated that in *TET2*-deficient mast cells, chronic activation via the oncogenic c-*KIT* D816V allele associated with mastocytosis, selects for a specific epigenetic signature characterized by hypermethylated DNA regions at immune response genes, suggesting a direct role of *TET2* in preventing immune tolerance in chronically activated mast cells, thus supporting it as a viable target to reprogram the innate immune response for innovative therapies (Rigo et al., 2022). However, the specific gene mutation identified in our patients (Y867H) is commonly found in CMML (Abdel-Wahab et al., 2009) and its role in pediatric mastocytosis is yet to be explored. The DCM patient carrying this mutation is indeed clinically stable and has normal tryptase levels.

In adults with mastocytosis, a high number of mutations generally indicates a worse prognosis (Martelli et al., 2020). Considering our findings, this does not appear to be the case for pediatric patients. All patients with more than one mutation had DCM, and only 25% of DCM cases tested negative on NGS, while negative genetic testing was more likely in MPCM (50%). This implies that DCM is the most associated type of CM with genetic alterations and is also the one in which multiple mutations are most likely to be found simultaneously. For instance, the two sisters, despite both having DCM, present different mutations (c-*KIT* Y553C and *SH2B3* L438V, independently). These mutations are likely sporadic and not genetically transmitted. The one harboring *c-KIT* Y553C mutation had disease onset at a few months of age, while the sister positive for *SH2B3* L438V had the first lesions at the age of seven. In the present analysis, no mutations in genes traditionally implicated in the adult setting, such as *RUNX1, DNMT3A, SRSF2, KRAS, NRAS*, and *SETD2* were identified. Conversely, in a recent study conducted by Sun et al. besides c-*KIT* mutations, 64 non-synonymous coding genetic mutations were found, including recurrently mutated genes (*ABL1, ATR, BCR, CBL, DNMT3A, FANCC, MSH2, NOTCH1, NTRK1, PDGFRB and RUNX1*). In their study, 86.7% of the pathogenic/likely pathogenic non-c-*KIT* mutations were detected in pediatric patients with SM. However, no mutations were identified in *SRSF2* or *ASXL1*, both high-risk genes associated with poor outcomes in adult SM (Sun et al., 2025).

To conclude, CM is more common in children, whereas systemic forms are rare. In the current study, c-*KIT* mutations resulted in 35% of the cases. The RT-PCR technique resulted more sensitive in finding c-*KIT* D816V, while NGS in detecting other mutations. *ASXL1, JAK2* and *TET2* mutations, detected in our population, are different to those reported in adult pts with SM. The presence of multiple mutations in the DCM form, does not appear to influence the evolution to a systemic form, unlike in adults. Until now, significant correlations between genetic status, as well as the immunophenotype of MCs in the skin, the disease phenotype and prognostication in pediatric mastocytosis have not been established (Ługowska-Umer et al., 2023). Multicenter, large-scale and long-term follow-up studies on childhood-onset mastocytosis are still required to clarify the foregoing aspects.

## Data Availability

The datasets presented in this study can be found in online repositories. The names of the repository/repositories and accession number(s) can be found in the article/Supplementary Material.
