# Supplementary material for: Molecular characterization of pediatric mastocytosis revealed different somatic mutations with uncertain prognostic value
Source: Front Cell Dev Biol. 2026 Feb 26;14:1780799. doi: 10.3389/fcell.2026.1780799 (PMC12979425; doi:10.3389/fcell.2026.1780799)
Supplement: Supplementary file 1 [file DataSheet1.pdf]

Supplementary Material

Supplementary Table 1. Summary of mutations detected by next-generation sequencing (NGS) technique in the examined cohort, including gene, nucleotide-amino acid changes and variant allele frequency (VAF) percentages.

Patient ID	Gene	Nucleotide alteration	Amino acid alteration	VAF (%)
**Mas_Ped03**	TET2	c.2599T>CT	p.Y867H	52
**Mas_Ped03**	LNK	c.622G>CG	p.E208Q	46
**Mas_Ped03**	KIT	c.2447A>T	p.D816V	<5
**Mas_Ped05**	NFE2	c.872T>CT	p.I291T	49
**Mas_Ped06**	KIT	c.2446G>T	p.D816Y	47
**Mas_Ped08**	KIT	c.2447A>T	p.D816V	0.16
**Mas_Ped10**	KIT	c.2447A>T	p.D816V	0.06
**Mas_Ped14**	KIT	c.2447A>T	p.D816V	0.06
**Mas_Ped19**	SH2B3	c.1145G>A	p.G382D	50
**Mas_Ped19**	ETV6	c.643G>C	p.A215P	50
**Mas_Ped21**	ASXL1	c.4189G>AG	p.G1397S	50
**Mas_Ped21**	JAK2	c.1177C>CG	p.L393V	52
**Mas_Ped25**	KIT	c.1658A>G	p.Y553C	23
**Mas_Ped26**	SH2B3	c.1312C>G	p.L438V	49
